# P-161. Assessing Antibiotic Usage Patterns, Knowledge Gaps, and Influencing Factors Among Poultry Producers, Veterinarians, and Farmers in Kerala, India

**DOI:** 10.1093/ofid/ofae631.366

**Published:** 2025-01-29

**Authors:** Jose J Kochuparambil

**Affiliations:** MQMH, Pala, Kerala, India

## Abstract

**Background:**

Antibiotic resistance is a pressing global concern exacerbated by widespread antibiotic use in livestock farming. This study aims to assess antibiotic consumption patterns and knowledge gaps among poultry producers, veterinarians, and farmers in Kerala, India, crucial for implementing effective antimicrobial stewardship strategies.

**Methods:**

A cross-sectional survey was conducted across Kerala, India, involving poultry producers, veterinarians, and farmers. Data were collected through structured interviews, focusing on antibiotic usage, knowledge, feeding practices, and biosecurity measures on poultry farms. Statistical analysis, including descriptive statistics, inferential tests, and regression analysis, was conducted to assess associations between variables and demographic factors.

**Results:**

Among the participants, 37% of poultry producers were from urban areas, while 63% were from rural areas. The majority of veterinarians (65%) had over five years of experience in poultry farming. Tetracyclines, aminoglycosides, and cephalexin were the most commonly used antibiotics, with 35% of producers reporting their frequent use. Regression analysis revealed that farm size and educational level significantly influenced antibiotic usage patterns, with larger farms and producers with higher education levels more likely to use antibiotics regularly (p < 0.001). Despite high antibiotic usage, there were significant knowledge gaps among stakeholders regarding antibiotic resistance. Only 37% of farmers were aware of the link between antibiotic use and antimicrobial resistance. A significant association between knowledge of antibiotic resistance and age was observed, with older farmers demonstrating higher levels of awareness. 57% of farmers above the age of 50 were aware of antibiotic resistance compared to 23% of farmers below the age of 30 (χ² = 15.6, p < 0.001). Additionally, farmers who had attended training programs on antibiotic stewardship were significantly more knowledgeable about antimicrobial resistance compared to those who had not received training (p < 0.01).

**Conclusion:**

The study highlights the need for targeted interventions to promote responsible antibiotic use and combat antimicrobial resistance.

**Disclosures:**

**All Authors**: No reported disclosures

